# Effect of different UCOE-promoter combinations in creation of engineered cell lines for the production of Factor VIII

**DOI:** 10.1186/1756-0500-4-178

**Published:** 2011-06-10

**Authors:** Ayyappan R Nair, Xie Jinger, Terry W Hermiston

**Affiliations:** 1 US Innovation Center, Bayer Healthcare, 455 Mission Bay Boulevard South, Suite 493, San Francisco, CA 94158, USA

## Abstract

**Background:**

The most common approach used in generating cell lines for the production of therapetic proteins relies on gene amplification induced by a drug resistance gene e. g., DHFR and glutamine synthetase. Practically, this results in screening large number of clones for the one that expresses high levels of the biologic in a stable manner. The inefficiency of mammalian vector systems to express proteins in a stable manner typically involves silencing of the exogenous gene resulting from modifications such as methylation of CpG DNA sequences, histone deacetylation and chromatin condensation. The use of un-methylated CpG island fragments from housekeeping genes referred to as UCOE (ubiquitous chromatin opening elements) in plasmid vectors is now well established for increased stability of transgene expression. However, few UCOE-promoter combinations have been studied to date and in this report we have tested 14 different combinations.

**Findings:**

In this report we describe studies with two different UCOEs (the 1.5 Kb human RNP fragment and the 3.2 Kb mouse RPS3 fragment) in combination with various promoters to express a large protein (B domain deleted factor VIII; BDD-FVIII) in a production cell line, BHK21. We show here that there are differences in expression of BDD-FVIII by the different UCOE-promoter combinations in both attached and serum free suspension adapted cells. In all cases, the 1.5 Kb human RNP UCOE performed better in expressing BDD-FVIII than their corresponding 3.2 Kb mouse RPS3 UCOE. Surprisingly, in certain scenarios described here, expression from a number of promoters was equivalent or higher than the commonly used and industry standard human CMV promoter.

**Conclusion:**

This study indicates that certain UCOE-promoter combinations are better than others in expressing the BDD-FVIII protein in a stable manner in BHK21 cells. An empirical study such as this is required to determine the best combination of UCOE-promoter in a vector for a particular production cell line.

## Findings

### Introduction

The production of a protein therapeutic depends on a number of different factors including expression, stability of the cell line, post translational modifications, protein trafficking, secretion and viability of cells when grown to high density in a bioreactor. The first step in creating a production cell line is the transfection of an expression vector that encodes the biologic. Levels of RNA expression depend on the expression vector used. Numerous commercially available mammalian expression vectors are available that utilize different regulatory elements to maximize the expression of the therapeutic protein including promoters and enhancers, post-transcriptional elements such as WPRE and sequences that control the termination of transcription. Besides the levels of protein expressed, achieving reliable and stable transgene expression in mammalian cells is another major challenge. The inefficiency of mammalian vector systems to express proteins in a stable manner typically involves silencing of the exogenous gene that results from modification of the integrated vector or its vicinity, such as methylation of CpG DNA sequences, histone deacetylation and chromatin condensation [1-3]. Most conventional vectors suffer from position effects and chromatin shut down thus resulting in gene silencing over time. Chromatin position effects makes the generation of mammalian cell lines expressing the protein therapeutic a difficult, time consuming and expensive process. The most commonly used approach used in the generation of such cell lines relies on gene amplification induced by a combination of a drug resistance gene e. g., DHFR and glutamine synthetase [4,5] and stringent selective pressure. The DHFR and glutamine synthetase (GS) genes are mainly used for selection in CHO and NSO cells, respectively. In both cases, the selection occurs in the absence of appropriate metabolite (s): glycine, hypoxanthine, and thymidine for DHFR and glutamine for GS. Both these markers have the advantage of supporting amplification of the copy number of the integrated DNA by exposure of selected cells to increasing amounts of methotrexate (MTX) or methionine sulphoximine (MSX), respectively [6]. Practically, this would result in screening a large number of clones for the one that expresses high levels of the biologic in a stable manner. There have been recent studies that have addressed the critical issue of stable expression. As an example, the inclusion of MAR (matrix associated regions) in the vector system has been exploited to increase the stability of expression of a number of exogenous genes in mammalian cells [7-10]. In addition, the discovery that certain un-methylated CpG sequences of housekeeping genes known as UCOE (ubiquitous chromatin opening elements) confers resistance to heterochromatin-mediated silencing has led to the incorporation of these elements into expression vectors including lentiviral vectors [11-13]. The addition of the UCOE in the expression vector is thought to create a transcriptionally active, open chromatin environment around an integrated transgene, maximizing its potential to be transcribed into protein and this effect is irrespective of the position of the transgene in the chromosome. The UCOE expression technology therefore provides major improvements in gene expression in stably-transfected mammalian cells through effects on the structure of chromatin (irrespective of chromosomal integration site) which results in the prevention of transgene silencing and gives consistent, stable and high-level gene expression. Therefore, by nature of the inherent properties of UCOE, the screening for cell lines that express high amounts of the biologic is less labor, time and cost intensive than other methods. Also, the chance of selecting a clone that is highly stable is better with plasmid vectors containing the UCOE.

There have been previous reports that large fragment containing the CpG islands from the human HNRPA2B1 and TBP-PSNB1 loci (designated RNP and TBP) are resistant to heterochromatin mediated silencing of endogenous genes transcribed from promoters associated with these genes [14]. The RNP UCOE is derived from and consists of the dual divergently transcribed promoter region of the HNRPA2B1-CBX3 genes, whereas the RPS3 UCOE consists of a single promoter element [15].

Also, a strong promoter, namely the human CMV promoter (hCMV) that is preceded by a large portion of the RNP CpG island (8 Kb) confers major benefits in transgene expression from this promoter when incorporated in a plasmid vector [16]. Reducing the CpG island fragment of the RNP locus to 1.5 Kb still conferred the benefits of expression and stability [16].

As far as we know, this is the first extensive study comparing different promoter-UCOE combinations has been undertaken. In this study, the 1.5 Kb RNP UCOE was compared to the 3.2 Kb mouse ribosomal protein S3 (RPS3) gene locus UCOE in the context of a number of different promoters including hCMV to express the transgene BDD-FVIII (B-domain deleted factor VIII) in the production line BHK21.

#### Comparison of the different UCOE-promoter combinations in adherent BHK21 cells

An earlier study compared different lengths of the human RNP CpG fragments in a vector system in the context of a strong promoter, *hCMV *(14). In that study, three different CpG fragment sizes were analyzed: 8.0 Kb, 4.0 Kb and 1.5 Kb. The 1.5 Kb fragment was able to confer stable high expression levels of transgene expression. Here, we constructed 7 different 1.5 Kb UCOE promoter combinations, and 7 different 3.2 Kb UCOE promoter combinations including the 1.5 Kb RNP UCOE with the hCMV promoter to drive the expression of BDD-FVIII in the production cell line BHK21.

**Figure 1:**
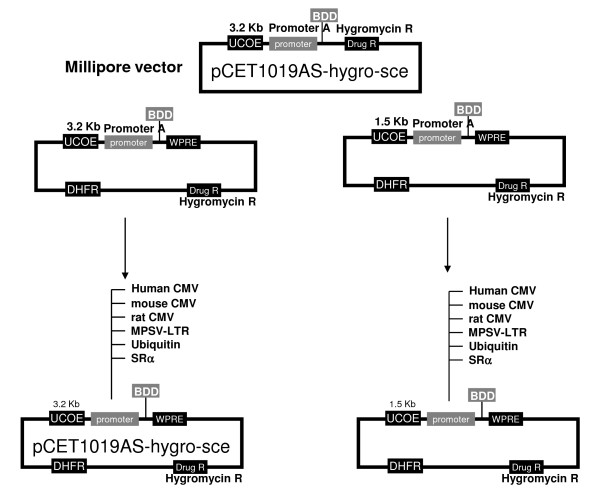
**Schematic representation of all the plasmid vectors constructed and used in the studies**.

**Figure 2:**
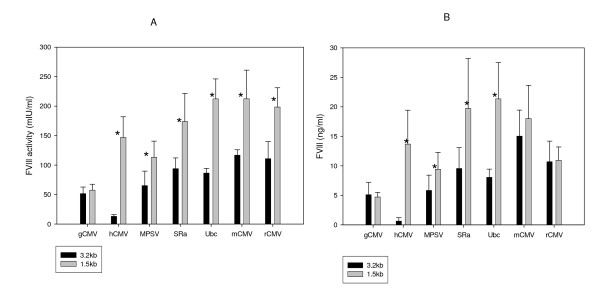
**Transient transfection studies in adherent BHK21 cells comparing the different UCOE plasmids**. Adherent BHK21 cells were transfected with equimolar amounts of the UCOE plasmids listed in figure 1 and the FVIII activity/ml (A), and the BDD protein levels secreted as measured by ELISA (B) were measured. Data was statistically analyzed using 't' test to detect significant differences between the 3.2 Kb and the 1.5 Kb UCOE containing expression constructs (*: p < 0.05 or more).

Fourteen constructs (Figure 1) were transiently transfected, and secreted BDD-FVIII was measured at the activity (Figure 2A) and the protein level (Figure 2B). As shown in Figure 2, there were many promoters that efficiently expressed active BDD-FVIII and were equal to or better than the hCMV promoter. Ubc and mouse CMV (mCMV) promoter driven expression was significantly higher than hCMV (with the 1.5 or 3.2 Kb UCOE, about 1.4-8 folds higher, P < 0.01 in activity and 1.1-25 folds higher in secreted protein levels, P < 0.05). Surprisingly, the expression levels driven by most promoters, except hCMV and MPSV promoters, were significantly greater than the guinea pig CMV (gCMV) promoter (with the 1.5 or 3.2 Kb UCOE, about 1.7-4 folds higher, P < 0.01 in activity and 1.6-4.5 folds higher, p < 0.01 in secreted protein levels). The general trend in BDD-FVIII activity/ml was comparable to the levels of protein secreted into the media and as measured by ELISA. In all cases, the 1.5 Kb human RNP UCOE-promoter combinations were better than the 3.2 Kb mouse RPS3 UCOE promoter combinations, suggesting the influence of the UCOE sequence on expression of the transgene (Figure 2). These UCOE-promoter combinations behaved differently in other cell lines tested; 293T, CHOK1 and HKB11 (data not shown).

**Figure 3:**
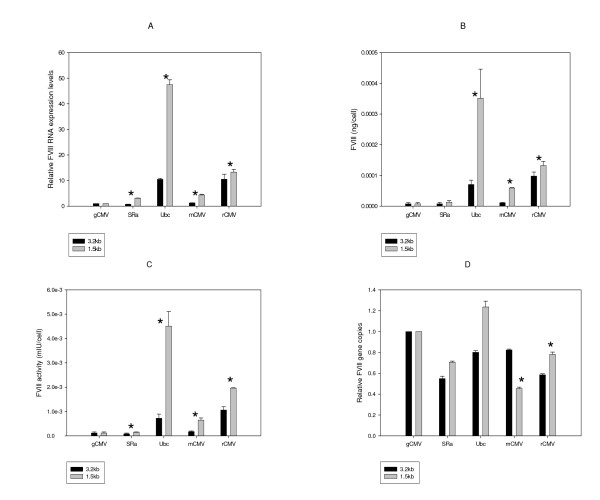
**Stable pooled adherent BHK21 studies**. Adherent BHK21 cells were transfected with different plasmids, cultured under hygromycin selection for one month and the levels of BDD mRNA (A), secreted protein (B) and secreted activity (C) were measured. The relative copies of integrated BDD cDNA was measured by qPCR (D). Data was statistically analyzed using 't' test to detect significant differences between the 3.2 Kb and the 1.5 Kb UCOE containing expression constructs (*: p < 0.05 or more).

The promoters that expressed the highest levels of BDD-FVIII in adherent BHK21 cells in transient transfection experiments were taken forward to create stable cell lines and compared with the *gCMV *promoter (in combination with both the 1.5 Kb human RNP and the 3.2 Kb mouse RPS3 UCOEs). One month post transfection, BDD-FVIII mRNA, secreted protein, and secreted activity levels were measured (Figure 3A-C). As with the transient transfection studies the 1.5 Kb UCOE always performed better than the 3.2 Kb UCOE to express BDD-FVIII (Figure 3A-C). The FVlll-BDD RNA, secreted protein and FVlll activity were increased about 1.27-4.5, 1.35-5.5 and 1.76-5.88 folds, respectively, with all of the tested promoters (P < 0.05-0.001). This indicates that the 1.5 Kb UCOE is a better choice of the two UCOE fragments evaluated in this study for maximal stable expression of BDD-FVIII in BHK21 cells. The 1.5 Kb human RNP in combination with the human ubiquitin promoter performed significantly better than the other UCOE-promoter combinations tested (Figure 3A-C); it showed over 40 fold higher expression of BDD-FVIII when compared to the 1.5 Kb RNP - *gCMV *promoter combination. To test whether this was an artifact caused as a result of differences in copies of integrated plasmids, a relative copy number determination was performed. Figure 3D demonstrates that the high levels of production and secretion of BDD-FVIII by the cell line transfected with the 1.5 Kb RNP-UCOE Ubiquitin promoter is not due to an unusually high number of integrated copies of that plasmid.

#### Comparison of the different UCOE-promoter combinations in serum free suspension adapted BHK21 cells

BHK21 cells were adapted to suspension culture by serum weaning so that they could be maintained in a serum free environment. The main advantage of growing these cells in serum free media is that the production cells are not in contact with animal proteins, and that would help purify the secreted therapeutic protein without any serum protein "contaminant". Even small levels of serum protein in the purified product will likely increase the chance of an immune response to the biologic in patients receiving treatment. The cells that were derived by the adaptation of BHK21 cells to suspension grew to a density of 4.6 × 10^6^/ml, were over 93% viable and had a doubling time of 20 hours in serum free media. The transfection of suspension adapted BHK21 cells in serum free media has traditionally been inefficient. Therefore in the past, to create a BHK21 production cell line required first the transfection of adherent cells, followed by generating stable lines as adherent cultures and then adapting these to serum free suspension culture. The advantage of transfecting serum free cells grown in suspension culture can not be underestimated as it saves time, effort and resources that ultimately results in reduced cost of goods.

**Figure 4:**
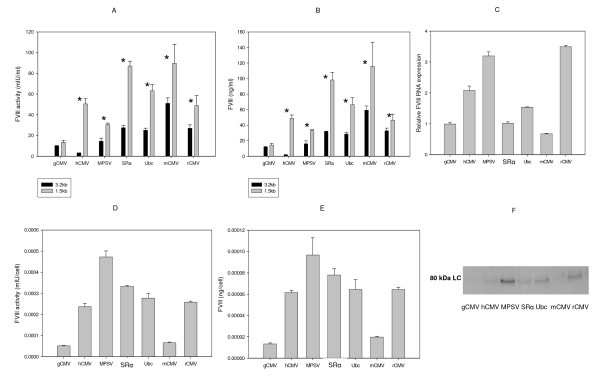
**Studies with serum free suspension adapted BHK21 cells comparing the different UCOE plasmids**. Suspension adapted BHK21 cells were transfected with equimolar amounts of the UCOE plasmids listed in figure 1 and the FVIII activity/ml (A), and the BDD protein levels secreted as measured by ELISA (B) were measured. After these cells were cultured under hygromycin selection for one month, the levels of BDD mRNA (C), secreted protein (D) and secreted activity (E) were measured. Data was statistically analyzed using 't' test to detect significant differences between the 3.2 Kb and the 1.5 Kb UCOE containing expression constructs (*: p < 0.05 or more).

**Figure 5:**
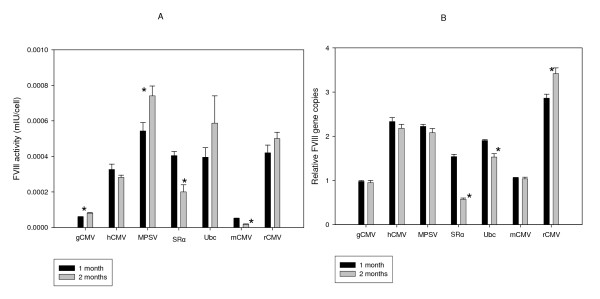
**Comparison of one and two month post transfection stability studies with serum free suspension adapted BHK21 cells**. Suspension adapted BHK21 cells were transfected with equimolar amounts of the UCOE plasmids listed in figure 1. These cells were cultured under hygromycin selection for one and two months. The levels of BDD activity (A) and relative BDD copy numbers were measured (B). Data was statistically analyzed using 't' test to detect significant differences between the 3.2 and the 1.5 UCOE containing expression constructs (*: p < 0.05 or more).

The data from transient transfection studies in serum free suspension adapted BHK21 cells using the same set of UCOE-promoter combinations as in the adherent studies are shown in Figure 4. Again as with adherent BHK21 cell studies, here too in all cases the1.5 Kb human RNP fragment- promoter combinations performed better in expressing the transgene when compared with the corresponding 3.2 Kb RSP3 mouse UCOE - promoter combinations. The expression levels increased 1.3-15.5 folds in activity and 1.2-25.5 folds in secreted protein with the tested promoters (P < 0.05-0.01, except gCMV). All the 1.5 Kb human RNP fragment - promoter combinations, except the gCMV, the MPSV promoters were equal to, or better expressers of BDD-FVIII than the 1.5 Kb human RNP-hCMV promoter combination (the expression levels driven by SRα, Ubc and mCMV were 1.3-15.7 folds higher in activities and 1.4-31 folds higher in secreted protein, Figure 4), As expected, the COATEST data that measures the activity of the secreted BDD-FVIII in the media (Figure 4A) correlated very well with the actual amount of protein in the media (Figure 4B). Due to the obvious superior expression from the 1.5 Kb human RNP constructs in both the adherent and suspension culture settings, all further studies were carried out only with the 1.5 Kb human RNP constructs. One month of culture under selective pressure with hygromycin was performed as with adherent culture. As was the case in adherent cell studies, there was a strong correlation between BDD-FVIII mRNA expression (Figure 4C, except in the case of the SRα and the rCMV promoters), secreted activity (Figure 4D) and secreted protein levels (as measured by both ELISA and western blot, Figures 4E and 4F, respectively). The surprising finding in this study was that the mCMV promoter driven expression had significantly dropped compared to what would be expected from transient expression studies, as also seen with the adherent stable cells. The integrated copy number studies suggest that this may partly be due to reduced integration of the plasmid (Figure 5B). The same reasoning could also be used for lower expression from the gCMV promoter in suspension cultured stable cells. Another deviation from what would be expected from transient expression studies was the high BDD-FVIII expression levels driven from the MPSV promoter; this promoter generated low levels of expression similar to the gCMV promoter in the transient transfection studies. The SRα, the Ubc and the *rCMV *promoter behaved as expected. The unusually high levels of expression and activity (> 40 fold higher than the gCMV promoter) seen from the Ubc promoter containing stable adherent cells was not seen in the suspension adapted cells.

#### Stability of the UCOE promoter derived cell lines

Serum free suspension BHK21 cells from the 1.5 Kb human RNP-promoter combinations were cultured for a period of two months to study the stability of these pooled populations. Activity (Figure 5A) and relative DNA copies (Figure 5B) were measured at one and two months post transfection. There was no significant difference in activity in most cases, except that there was a reduction in the case of the *mCMV *and SRα promoter based cell line and a slight increase in activity in the case of the MPSV promoter (Figure 5A). The reduction in activity in SRα promoter cell line may be attributed to the reduction in relative DNA integrated copies, and the reduction in the mCMV promoter cell line seems not to be due to a reduction in copy number, but due to some post integration phenomenon (Figure 5B). Recently it has been shown that the ability of UCOE to confer stability of expression on a linked promoter is due at least in part to it being able to negate DNA-methylation mediated silencing [13,15], and this may contribute towards the observed stability in most of the UCOE-promoter combinations tested but not all (loss of stability in the case of mCMV and SRa).

## Conclusion

The 1.5 Kb human RNP CpG fragment has previously been shown to confer higher expression and stability of the transgene in production cells when placed ahead of the hCMV promoter. We investigated two UCOE fragments, the 1.5 Kb human RNP CpG fragment and the 3.2 Kb mouse RPS3 fragment. These UCOE fragments were placed upstream of seven different commonly used promoters to generate 14 different plasmid vectors that encode the FVIII-BDD cDNA to study the effect of the UCOE sequence and the combination of the different promoters to express FVIII-BDD in BHK21 cells.

We observed that in combination with the different promoters, the corresponding 1.5 Kb UCOE is better than the 3.2 Kb UCOE at expressing FVIII-BDD in both attached and serum free suspension adapted BHK21 cells. The differential integration of the 1.5 Kb UCOE and the 3.2 Kb UCOE plasmids can not be attributed to this as the relative copy numbers of integrated plasmids in pooled populations of established cells were not sufficiently different to explain the differences in expression by the two UCOEs. The dual divergently transcribed promoter of the HNRPA2B1-CBX3 genes may contribute towards this phenomenon. Based on this, we conclude that the 1.5 Kb RNP fragment is a better choice for the expression of the FVIII-BDD transgene in BHK21 cells.

From transient transfection studies we conclude that in both adherent and suspension adapted BHK21 cells, all the promoters were better than the gCMV promoter, except the hCMV promoter in the context of the 1.5 Kb UCOE is better than the corresponding gCMV promoter. The similarities in the expression trends between suspension adapted and adherent BHK21 cells suggests that the transcriptional machinery is similar in the two morphologically different cell types that were derived from the same parental BHK21 cells. However, we observed differences in expression level trends when these plasmids were transfected into other cell types.

When the transfected cells were cultured for over a month, there were many unexpected observations. In the case of adherent cells, there was a substantial loss of expression from the mCMV and the SRα promoters when compared to the gCMV promoter and the Ubc promoter based expression seemed to increase relative to the gCMV promoter. These changes were not due to large differences in copy numbers. The decrease in expression levels may be explained by DNA methylation mediated gene silencing over time. It is however difficult to explain the increased expression by the Ubc promoter. Suspension adapted cells grown for a month showed differences in gene expression levels, but these may be a result of differences in gene copy numbers. These cells are stable as the activity levels nor the gene copies changed after an additional month of continuous culture under selection. There was no silencing observed. The only significant change was decreased expression from the SRα promoter and that can be explained as a result of decreased number of integrated copies that may be a result of shift in population pools.

We conclude that an empirical study such as this is necessary to evaluate the expression vector system for a given production cell line. Also, optimizing conditions for transfection in suspension adapted serum free BHK21 cells can be achieved and is beneficial in not only saving time and resources, but also in the purification of the biologic with fewer protein contaminants resulting in reduced immunogenicity.

## Competing interests

The authors declare that they have no competing interests.

## Authors' contributions

ARN conceived of the studies, designed experiments, prepared the manuscript and monitored the progress of the studies. JX carried out the studies, performed statistical analysis and was involved in preparing the manuscript. TWH conceived of the studies, monitored the progress, and was involved in the preparation of the manuscript.
